# Low-Dose Resveratrol Inhibits RIPK3-Mediated Necroptosis and Delays the Onset of Age-Related Hearing Loss

**DOI:** 10.3389/fphar.2022.910308

**Published:** 2022-06-29

**Authors:** Zeyin Yang, Yan Zhang, Shuling Yang, Yongqing Ding, Yan Qu

**Affiliations:** ^1^ Department of Otolaryngology, The Third Hospital of Hebei Medical University, Shijiazhuang, China; ^2^ ENT & HN Surgery Department, Shijiazhuang People’s Hospital, Shijiazhuang, China; ^3^ Department of Otolaryngology, Tangshan People’s Hospital, Tangshan, China; ^4^ Animal Laboratory, The Third Hospital of Hebei Medical University, Shijiazhuang, China

**Keywords:** age-related hearing loss, presbycusis, resveratrol, necroptosis, ripk3, aging cochlea

## Abstract

**Background:** To investigate the pathophysiology of age-related hearing loss (ARHL) and the mechanism of resveratrol (RSV) in prevention and treatment of ARHL.

**Methods:** C57BL/6 mice of different ages were used in this study. Auditory brainstem response (ABR) was performed to assess hearing and identify abnormalities. Surface preparation and hair cell-specific marker Myo VIIa were employed to evaluated cochlear hair cell losses. Scanning electron microscopy (SEM) was to observe the microstructure of the organ of Corti (OC). The expression of related proteins in the RIPK1/RIPK3/MLKL pathway in cochlear tissue was detected by immunofluorescence.

**Results:** In old mice (15 months), the ABR threshold increased significantly compared with the young mice. After 50 mg/kg RSV intervention, the hearing threshold of the old mice was significantly reduced at 8 kHz and 12 kHz as well as click. 100 mg/kg RSV led to a statistically significant reduction in hearing threshold only at clicks, whereas 300 mg/kg RSV showed no difference at all frequencies tested. In terms of cochlear hair cell loss, the damage of OHC and IHC was severe in old mice, but the damage was evidently reduced in RSV 50 mg/kg group. Notably, in the RSV 300 mg/kg group, the loss and disorientation of both the OHCs and IHCs were aggravated. Under SEM, a large number of OHCs were lost in the old group, but increased significantly in the RSV 50 mg/kg group, and even the OHCs were more seriously damaged in the RSV 300 mg/kg group. Furthermore, immunofluorescence showed that 50 mg/kg RSV significantly reduced the expression of RIPK3, RIPK1, and MLKL in the cochlea during aging, especially in necroptosis-sensitive regions OCs and SGN.

**Conclusion:** Low-dose RSV inhibited RIPK3-mediated necroptosis in aging cochlea and delayed the onset of ARHL, which was a promising therapeutic strategy for ARHL.

## Introduction

Age-related hearing loss (ARHL), also known as presbycusis, is a progressive, irreversible and symmetrical bilateral neurosensory hearing loss disorder caused by cochlear degeneration or the loss of auditory nerve fibers during cochlear aging (Chern and Golub, 2019). Decreased hearing threshold sensitivity, impaired speech discrimination, slowed central acoustic signal processing, and impaired sound source localization are the most striking features of ARHL (Gates and Mills, 2005). With the gradual aging of society, the incidence of ARHL is getting higher and higher. More than 10% of the world’s population, especially those aged 65 and older, is already affected by disabling hearing loss. It is estimated that by 2025, 1.2 billion people worldwide will be over the age of 60, of which more than 500 million will suffer severe presbyage-related deafness (Huang and Tang, 2010). Presbycusis is an extremely complex multifactorial process, which is influenced by many factors, such as cochlear aging, environmental factors and genetic factors. Those with untreated presbycusis often present with depression, loss of self-esteem and even dementia (Woodcock and Pole, 2008). Unfortunately, despite considerable efforts and achievements, there is still no effective cure for ARHL patients, and its treatment prognosis is not completely satisfactory as the mechanisms underlying are not well understood (Fujimoto and Yamasoba, 2014).

Necroptosis is an accidental and unregulated, passive cell death induced by nonspecific and nonphysiological stress (Wu and Ye, 2020). Due to the absence of a trigger, necrosis is generally classified as a negative factor, with no markers of apoptosis or autophagy. Today, necrosis is morphologically characterized by increased cell volume, distended organelles, disruption of the plasma membrane, and subsequent loss of cellular contents (Lyu et al., 2020). This biological process is activated by the receptor-interacting serine/threonine protein kinase (RIPK) homologous interaction motif (RHIM) and is mediated by the formation of a RIPK1/RIPK3 complex, which exhibits necrosis-like morphological characteristics (Liao et al., 2020). Currently, necroptosis has been highlighted for its increasingly critical role in the pathogenesis of neurodegenerative diseases, aging, and hearing impairment, including cisplatin and aminoglycoside-induced ototoxicity and noise-induced hearing loss (Zhang et al., 2017). However, in ARHL *in vivo* model, the necroptosis of cochlear has rarely been and explored and described. Here, we aimed to focus on necroptosis to investigate the pathophysiology of ARHL, which may be beneficial for the discovery of clinically applicable drugs.

Resveratrol (3,5,4′-trihydroxystilbene; RSV), a natural phytoalexin and polyphenol, exists in numerous genera, including grapes, mulberries, peanuts and rhubarbs (Malaguarnera, 2019). RSV is involved in a variety of biological processes and plays a crucial part in anti-aging, anti-tumor and cardioprotective effects (Singh et al., 2019; Tian and Liu, 2020). In addition, it also plays an excellent protective role in the treatment of noise, cisplatin and aminoglycoside-induced hearing loss (García-Alcántara et al., 2018; Li et al., 2019; Lee et al., 2020). Notably, available data suggested that RSV has a beneficial effect on presbycusis, (Su et al., 2019; Xiong et al., 2019), especially when it begins before hearing loss begins (Muderris et al., 2022). Although the underlying mechanisms of these otoprotective effects of RSV still remain undetermined, restoration of autophagy (Pang et al., 2019), amelioration of oxidative stress and inflammation may be the plausible mechanisms. The effects of RSV vary depending on its dose and bioavailability. Low-dose RSV was shown to have significant anti-apoptotic and cardioprotective functions, whereas high-dose RSV conversely promoted apoptosis and vascular endothelial injury (Mukherjee et al., 2010). Additionally, an *in vivo* cisplatin ototoxicity study on rat revealed that RSV exerted hearing protection at low doses of 0.1 mg/kg/day, whereas RSV enhanced ototoxicity at high doses of 1 or 10 mg/kg/day (Olgun et al., 2014). Therefore, we speculated that there exists a dose range of otoprotective RSV in presbycusis, and further excavating the possible protective mechanism of RSV will bring us closer to finding a clinical solution for ARHL.

Hence, in this study, C57BL/6 mice at different ages were used to construct ARHL model, to investigate the pathophysiology of presbycusis and the mechanism of RSV in prevention and treatment of ARHL. Our findings may provide novel therapeutic targets for the prevention and treatment of ARHL.

## Materials and Methods

### Animals and Groups

Totally, 100 male C57BL/6 mice purchased from Beijing HFK Bioscience Co., Ltd. (Beijing, China) were employed in this study. All mice were housed in an air-conditioned animal facility with a constant temperature of 23°C, with water and food provided *ad libitum* and maintained under a 12 h light/dark circle. After a week of adaptation, the animals were randomly divided into five groups: I) young group (3 months of age, *n* = 20), II) old group (15 months of age, N = 20), III) old + 50 mg/kg/d RSV (RSV 50 mg/kg, *n* = 20) group, IV) old + 100 mg/kg/d RSV (RSV 100 mg/kg, *n* = 20) group, and V) old + 300 mg/kg/d RSV (RSV 300 mg/kg, *n* = 20) group. In the young and old groups, the mice were fed for standard chow; In the old + RSV groups, the mice were subjected to dietary supplementation with RSV (50 mg/kg/d RSV, 100 mg/kg/d RSV, 300 mg/kg/d RSV)added to the chow for a period of 12 months from 3 months of age. The animal experiment was approved by the Animal Ethics Committee of The third Hospital of Hebei Medical University and complied with the International Laboratory Animal Management Regulations.

### Auditory Brainstem Response (ABR)

Animals were anesthetized with 90 mg/kg ketamine and 10 mg/kg xylazine mixture to measure the auditory-evoked potentials. The TDT System-3 (Tucker Davis Technologies, Gainesville, FL, United States) hardware and software were used to obtain the ABR results. Measurements were performed in a sound-proofed room to ensure minimization of background noise. The reference and ground electrodes were inserted at the vertex and contralateral thigh, and the measuring electrode was placed at the ipsilateral retroauricular area.

Ten millisecond (ms) tone bursts with a 1 ms rise/fall time were presented at 4,8,12, 16, 24, and 32 kHz at a rate of 21.1/s. By reducing the sound intensity in 5 dB intervals around the threshold, the average responses to 1,000 stimuli were obtained. All ABR recordings were performed by the same investigator and ABR evaluations were assigned to specialists blinded to the treatment conditions.

### Surface Preparation

After intraperitoneal injection of a mixture of ketamine (90 mg/kg) and xylazine (10 mg/kg), the animals were decapitated and the cochleae were dissected in phosphate-buffered saline (PBS). A small hole with a diameter of 0.5 mm was drilled in the cochlea apex, the stapes were removed, and a slit in the round window was made to gently circulate 10% paraformaldehyde in PBS through the apex to the basal turn with a pipette under a microscope. All specimens (*n* = 6) were immersed in 10% paraformaldehyde in PBS overnight, rinsed with 0.1 M PBS three times and maintained in 10% EDTA in PBS for 4 d. Afterwards, the basilar membrane was dissected from the cochlea into segments of two-thirds of the turns and stained with TRITC-conjugated phalloidin (Sigma, P1951). Finally, the segments were mounted on a slide in glycerol as a surface preparation.

### Immunofluorescence of Cochlear Surface Preparations and Cryosections

In short, prepared cochlear sections (*n* = 6) were first incubated in PBST (Beijing Xuyang Chemical Technology Research Institute Co., Ltd., China) for 1 h at room temperature. After washed with PBS and blocked with 5% goat serum, the specimens were incubated overnight at 4°C with following primary antibodies: anti-myosin VIIa (25–6790, rabbit anti-mouse polyclonal antibody, 1:200, western blot, immunohistochemistry, immunocytochemistry, immunofluorescence, Proteus BioSciences, Ramona, CA), anti-RIPK3 (A5431, rabbit anti-mouse polyclonal antibody, 1:100, western blot, immunohistochemistry, immunofluorescence, Abclonal, UAS), anti-MLKL (A5579, rabbit anti-mouse polyclonal antibody, 1:100, western blot, immunohistochemistry, immunofluorescence, Abclonal, UAS), anti-RIPK1 at 1:100 (610458, mouse anti-mouse monoclonal antibody, 1:100, western blot, immunohistochemistry, immunofluorescence, immunoprecipitation, BD Biosciences, United States) followed by anti-Alexa flour 488-labeled IgG (A32723, goat anti-mouse polyclonal antibody, 1:200, western blot, immunocytochemistry, immunofluorescence, Invitrogen, United States) or anti-Alexa flour 594-labeled IgG (A48284, goat anti-rabbit polyclonal antibody, 1:200, immunocytochemistry, immunofluorescence, Invitrogen, United States). In the control, the primary antibodies were replaced with PBS. Finally, the samples in different groups were counterstained with DAPI in the dark and observed a confocal microscope (Zeiss LSM 710, Germany).

### Scanning Electron Microscopy (SEM)

Following final ABR recordings, the deeply anesthetized mice were decapitated and the cochleae (*n* = 6) were washed with PBS gently to remove the blood and hair. Then the samples were fixed in 2.5% phosphate-buffered glutaraldehyde (Shaanxi Dideu Medichem Co. Ltd., China) for 20 min at 37°C and post-fixed in 1% osmium tetroxide (Sigma-Aldrich Co. LLC., United States) in water at 37°C for 30 min. Following gradient dehydration for each time 15 min with ethanol and acetone (50% ethanol; 70% ethanol; 90% ethanol; 90% acetone; 100% acetone) at 37°C, the samples were dried with critical point dryer in a liquid CO_2_ and were attached to metal stubs using carbon stickers and sputter-coated with gold for 30 s. Finally, the samples were observed and the images were taken with a SEM (JSM-5310; JEOL, Ltd., Tokyo, Japan).

### Statistical Analysis

Data were analyzed using IBM SPSS Statistics Premium V21. Kruskal–Wallis rank sum test was employed to determine statistically significant differences in ABR thresholds across groups. *p* < 0.05 was regard as statistically significant.

## Results

### Low-Dose RSV Evidently Reduced the ABR Thresholds After RSV Administration

**FIGURE 1:**
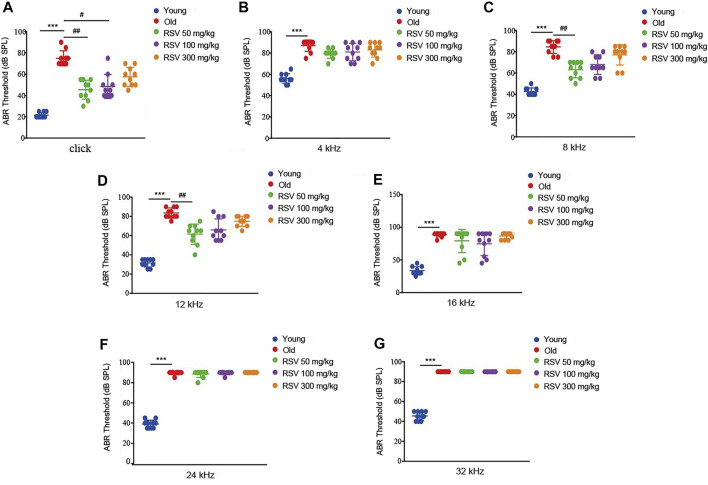
Low-dose RSV evidently reduced the ABR thresholds after RSV administration. **(A)** The ABR thresholds under click sound; **(B)** The ABR thresholds under frequency of 4 kHz; **(C)**. The ABR thresholds under frequency of 8 kHz; **(D)** The ABR thresholds under frequency of 12 kHz; **(E)** The ABR thresholds under frequency of 16 kHz; **(F)** The ABR thresholds under frequency of 24 kHz; **(G)** The ABR thresholds under frequency of 32 kHz. RSV, resveratrol; ABR, auditory brainstem response. ***p < 0.0001 vs. Yong group; #*p* < 0.05, ##*p* < 0.001 vs. Old group.

ABR determines whether the auditory pathway is normal by judging various parameters of the waveform. First, age-related functional impairment of hearing in male C57BL/6 mice aged 3 and 15 months were detected. As shown in Figure 1, the mice in the old group exhibited significantly increased ABR thresholds at 4, 8, 12, 16, 24, and 32 kHz and when click sounds were delivered, indicating that old C57BL/6 mice developed an obvious hearing loss. Intriguingly, the hearing thresholds of RSV 50 mg/kg group were evidently lower than those of the old group at 8 kHz and 12 kHz, also the click sounds after administration of RSV for 12 months (Figures 1A,C,D). In addition, compared with the old group, the threshold drift at 4 kHz in the RSV 50 mg/kg group represented a downward trend, but there was no statistical significance between the two groups (Figure 1B). Threshold shifts at 16, 24 and 32 kHz showed no difference between the above two groups (Figures 1E–G). In the RSV 100 mg/kg group, we observed that the hearing thresholds at 8 and 12 kHz were decreased after RSV administration, but there was no statistical difference compared with the old group. While when click sounds were delivered, the RSV 100 mg/kg group exhibited a statistically significant hearing threshold reduction (Figure 1A). Thresholds across all tested frequencies showed no difference in the RSV 300 mg/kg group.

### Low-Dose RSV Evidently Reduced Hair Cell Loss During the Progression of ARHL

**FIGURE 2:**
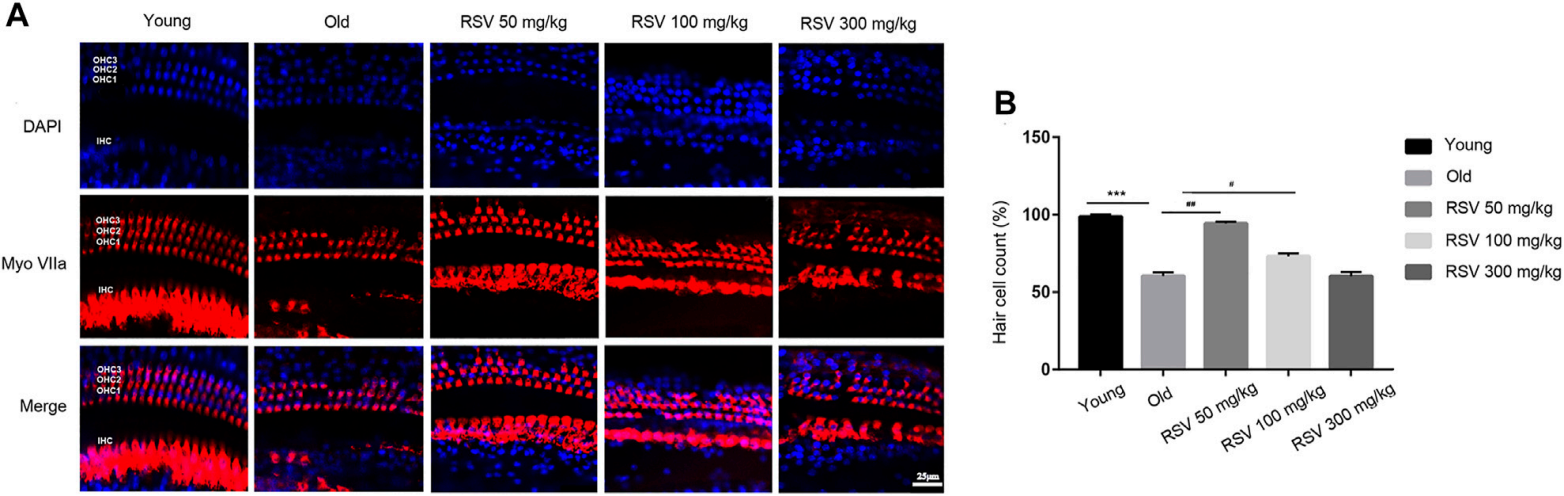
Low-dose RSV evidently reduced hair cell loss during the progression of ARHL. **(A)** Tissues were double-stained with DAPI (blue) and myosin VIIa (red) to visualize the hair cells with one row of IHCs and three rows of OHCs; Scale bar = 25 μm; **(B)** Quantitative analysis of hair cell number in each group. RSV, resveratrol; ARHL, age-related hearing loss; IHCs, inner hair cells; OHCs, outer hair cells. ***p < 0.0001 vs. Yong group; #*p* < 0.05, ##*p* < 0.001 vs. Old group.

Cochlear hair cell losses were first evaluated using a surface preparation. In young mice, the surface preparation exhibited a regular outlines, manifesting as one row of inner hair cells (IHC) and three rows of outer hair cells (OHC). Cochlear whole-mount examination revealed that both OHCs and IHCs were more severely damaged in old mice. Aging caused disorientation and reduction of the OHCs, however, damage to the OHCs was more prominent than the damage to IHCs. These hair cell injuries were attenuated in the RSV 50 mg/kg group, but it was not obvious in the RSV 100 mg/kg group. Notably, in the RSV 300 mg/kg group, the loss and disorientation of both the OHCs and IHCs were aggravated (Figure 2A). Correspondingly, we counted the number of hair cells in each group. As shown in Figure 2B, the number of hair cells was significantly reduced in Old group compared with Young group. Interestingly, the 50 mg/kg RSV intervention evidently increased cell number, but this increase was not evident at 100 mg/kg RSV and 300 mg/kg RSV.

**FIGURE 3:**
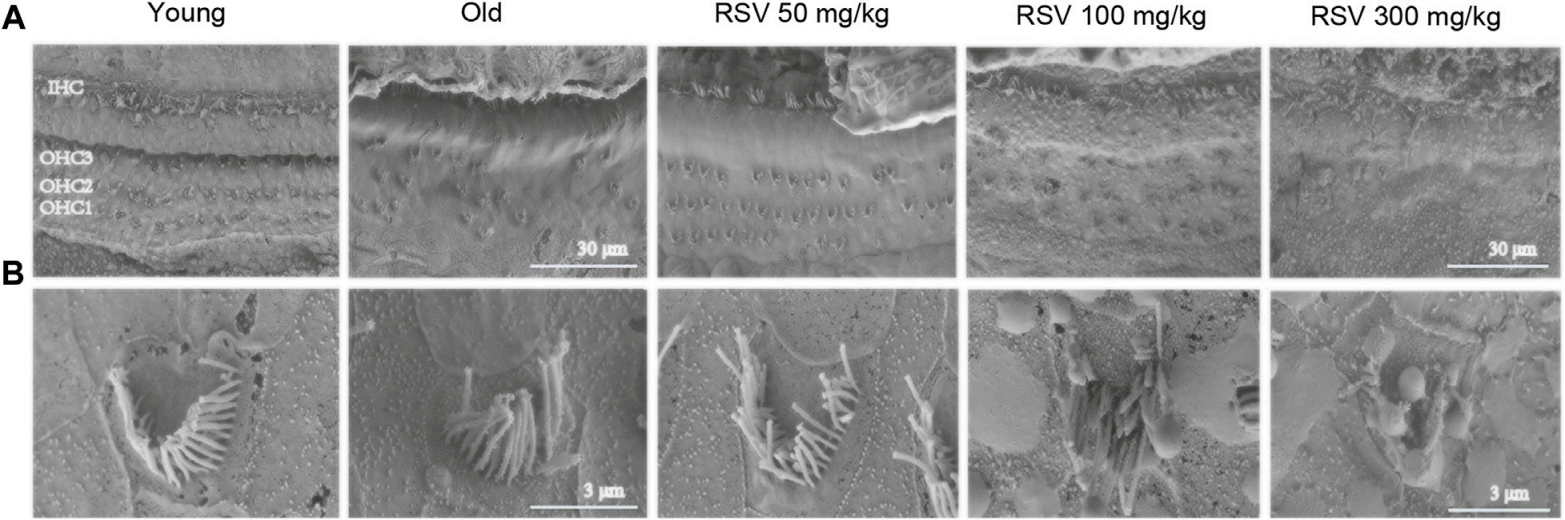
Low-dose RSV evidently reduced hair cell loss during the progression of ARHL. **(A,B)** The SEM of OC in different groups (Yang group, Old group, RSV 50 mg/kg group, RSV 100 mg/kg group and RSV 300 mg/kg group); Scale bar = 30 μm/3 μm; In the Old group, the RSV 100 mg/kg and the RSV 300 mg/kg groups, the V-shape of cells were disappeared, the arrangement were disordered and lodging and the number was decreased evidently. While in young and RSV 50 mg/kg, stereocilia bundles show a V-shape, neatly arranged and slightly absent; RSV, resveratrol; OHCs, outer hair cells; OC, organ of Corti.

SEM showed the narrow, linear shape of IHC stereocilial bundles and V-shape of OHC stereocilium (Figure 3). In the young group, the SEM displayed an overview of the organ of Corti (OC), with three rows of OHCs and one row of IHCs, nearly without loss of hair cells. However, a large number of OHCs were lost in the old group. Notably, the number of OHCs was increased significantly in the RSV 50 mg/kg group, but not in the RSV 100 mg/kg group and even seriously damaged in the RSV 300 mg/kg group, including the loss and morphological changes (Figure 3A). Moreover, increased magnification SEM showed more details of the stereocilia bundles on OHCs. To be specific, in the old, the RSV 100 mg/kg and the RSV 300 mg/kg groups, the V-shape were disappeared, the arrangement of were disordered and lodging and the number was decreased evidently. While in young and RSV 50 mg/kg, stereocilia bundles show a V-shape, neatly arranged and slightly absent (Figure 3B).

### Low-Dose RSV Evidently Inhibited RIPK3-Mediated Necroptosis in Aging Cochlea

**FIGURE 4:**
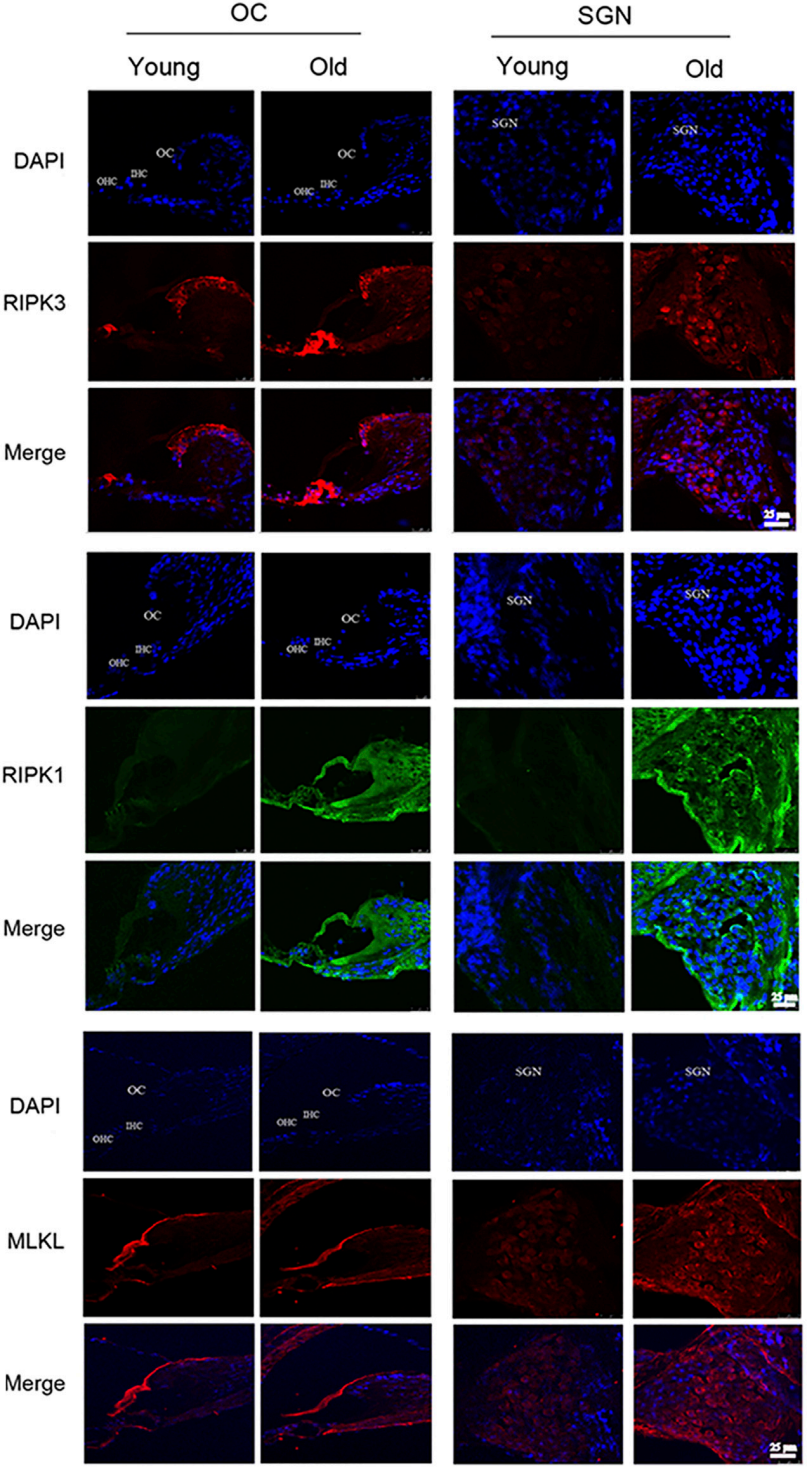
Low-dose RSV evidently inhibited RIPK3-mediated necroptosis in aging cochlea. The expression of RIPK3, RIPK1 and MLKL at different locations (OC and SGN) in Young and Old groups was detected by immunofluorescence; RSV, resveratrol; RIPK3, receptor-interacting serine/threonine protein kinase 3; RIPK1, receptor-interacting serine/threonine protein kinase 1; MLKL, mixed lineage kinase domain-like protein; OC, organ of Corti; SGN, spiral ganglion neuron. Scale bar = 25 μm.

**FIGURE 5:**
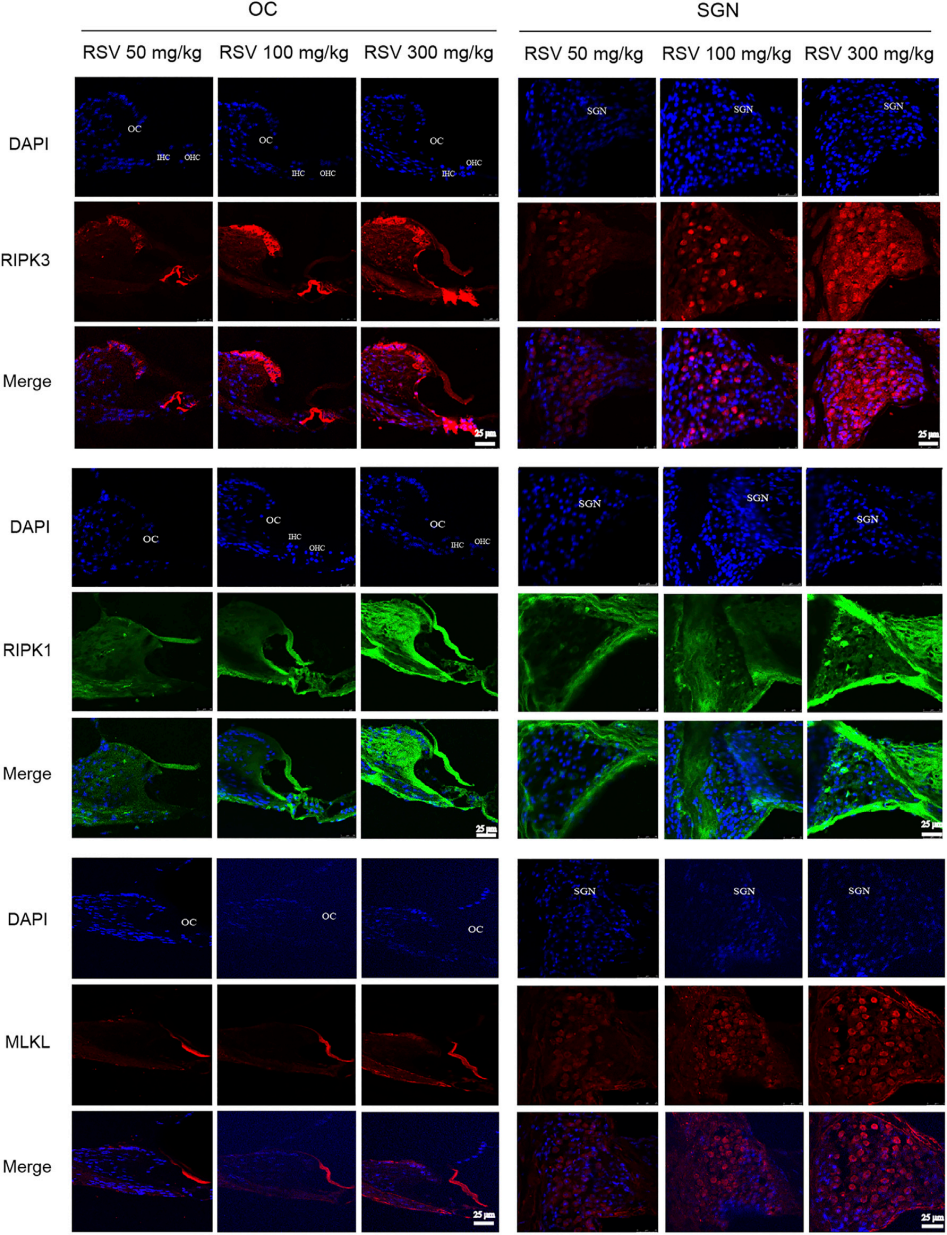
Low-dose RSV evidently inhibited RIPK3-mediated necroptosis in aging cochlea. The expression of RIPK3, RIPK1, and MLKL at different locations (OC and SGN) in RSV 50 mg/kg, RSV 100 mg/kg and RSV 300 mg/kg groups was detected by immunofluorescence; RSV, resveratrol; RIPK3, receptor-interacting serine/threonine protein kinase 3; RIPK1, receptor-interacting serine/threonine protein kinase 1; MLKL, mixed lineage kinase domain-like protein; OC, organ of Corti; SGN, spiral ganglion neuron. Scale bar = 25 μm.

**FIGURE 6:**
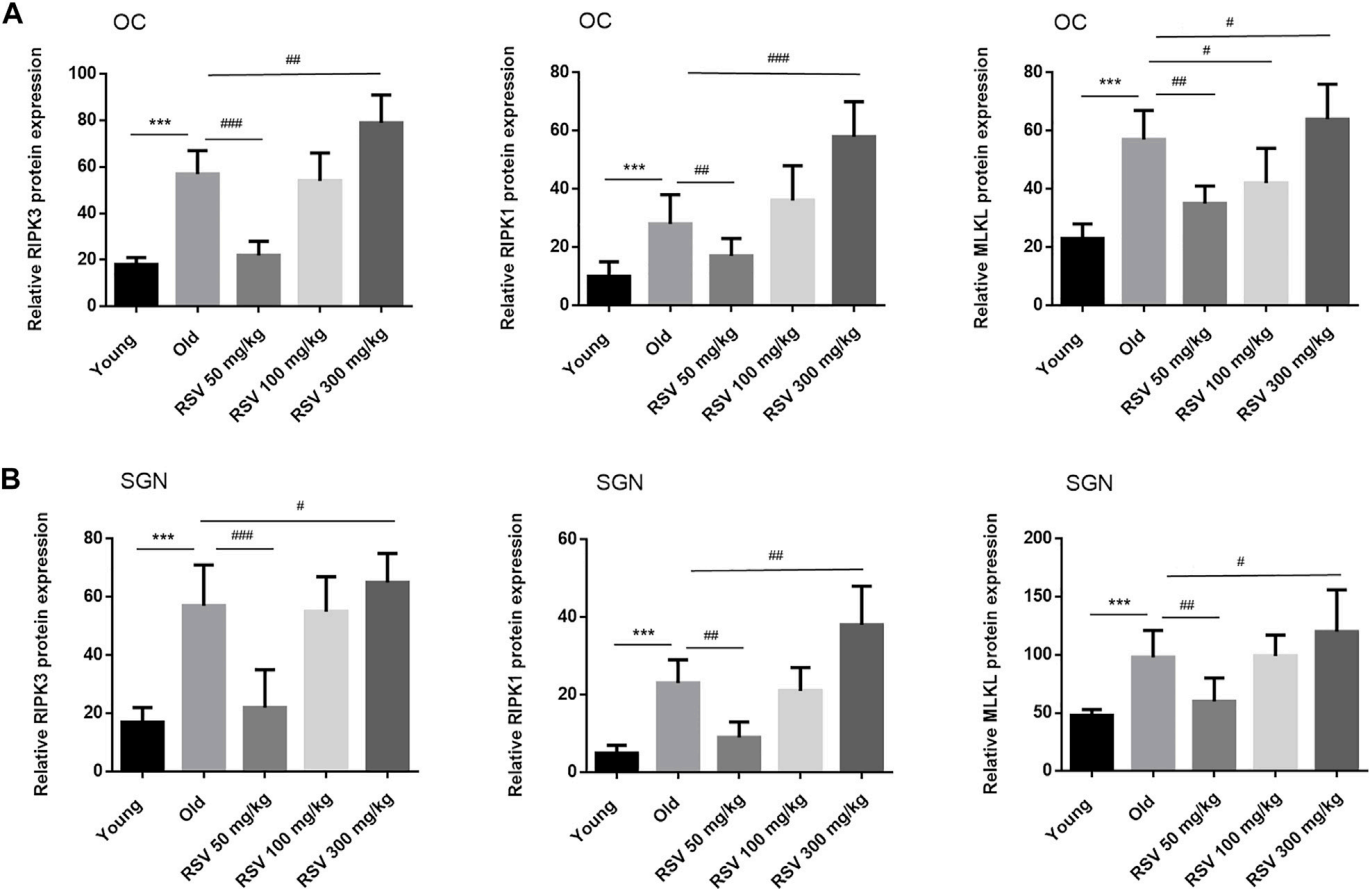
Low-dose RSV evidently inhibited RIPK3-mediated necroptosis in aging cochlea. Quantitative detection of RIPK3, RIPK1, and MLKL proteins in different groups of mice; ***p < 0.0001 vs. Yong group; #*p* < 0.05, ##*p* < 0.001, and ##*p* < 0.0001 vs. Old group. RSV, resveratrol; RIPK3, receptor-interacting serine/threonine protein kinase 3; RIPK1, receptor-interacting serine/threonine protein kinase 1; MLKL, mixed lineage kinase domain-like protein; OC, organ of Corti; SGN, spiral ganglion neuron.

The most defined molecular pathway of necroptosis is mediated through RIPK1/RIPK3/MLKL pathway (Chen et al., 2017). To further explore the role of RSV and RIPK1/RIPK3/MLKL in ARHL, we examined the expression of pathway-related proteins in cochlea tissues. In Figure 4, we observed that RIPK3, RIPK1 and MLKL were prominent in the cochlear in old mice compared with young mice, especially in OC and spiral ganglion neuron (SGN). Consistently, the quantitative data showed the same expression trend (Figure 6). These results suggested that RIPK3-dependent necroptosis was promoted in cochlear tissues during aging, and OCs and SGNs might be the sensitive areas to necroptosis. Further, we compared the changes of the above proteins after RSV intervention. As depicted in Figure 5, compared with those the mice in the old group, the protein expression of RIPK1, RIPK3 and MLKL were remarkably decreased in the RSV 50 mg/kg group, but not significantly changed in RSV 100 mg/kg group, and even increased in RSV 300 mg/kg group. The above protein changes can be further observed in the quantitative data (Figure 6). These data suggested that RSV inhibited the RIPK1/RIPK3/MLKL pathway and the necroptosis in aging cochleae in dose-dependent manners. Specifically, low-dose RSV had partial otoprotective effects in ARHL, whereas high-dose RSV did not ameliorate age-related hearing impairment.

## Discussion

In the present study, we demonstrated that long term (12 months) administration of low-dose RSV partially decreased the auditory threshold shifts in C57BL/6 mice with ARHL, as well as the cochlear hair cells were protected during the process of aging. We also identified elevated RIPK3 in aging cochleae, particularly in the OC and SGN. Moreover, RSV attenuated the expression of RIPK1, RIPK3, and MLKL in the aging cochlea. Therefore, RSV appears to protect the cochlea hair cells from senescence damage and delay the onset of ARHL by inhibiting RIPK3-mediated necroptosis. This study contributed to the existing body of knowledge in this field by exploring the dose-dependent otoprotective effects of RSV on underlying molecules mechanism.

With the in-depth research and excavation of ARLH, the molecular mechanism related to ARHL and cochlear cell degeneration has been understood to a certain. Age-related changes in the functional components of the inner ear have been reported to affect many biological processes, involving oxidative stress, mitochondrial DNA mutations, DNA damage, cell apoptosis, etc (Menardo et al., 2012). When aging occurs, necrosis, apoptosis and autophagy can cause death of cochlear hair cells and spiral ganglion neurons. In the cochlear aging model of CBA mice, the expression of some apoptosis-related genes changes with age and hearing loss, so apoptosis may play an irreplaceable role in ARHL (Tadros et al., 2008). Different from apoptosis, necrotic apoptosis, as another programmed cell death, shows morphological characteristics similar to necrosis. They may exist in cells at the same time (Kurabi et al., 2017). As reported, RSV could compete with cell apoptosis, exerting an otoprotective function (Liu et al., 2021). In addition, RSV has also been shown to have beneficial effects in ARHL, especially when administered prior to the onset of hearing loss (Muderris et al., 2022). However, existing data have not shown whether RSV can alleviate necroptosis in the aging cochlea. Thus, here, ARHL model was constructed in C57BL/6 mice to e explore the effect of RSV on necroptosis. C57BL/6 J mice had a short life span, well-defined genetics, and early onset hearing loss lasted for a lifetime, which better simulated the reality of human hearing loss.

In this study, we found that aged mice exhibited greater hearing loss, whereas daily RSV administration was able to significantly reduce ARHL manifestation in C57BL/6 mice. Furthermore, it is worth noting that this benefit was most pronounced in the low-dose group of the RSV 50 mg/kg group, characterized by a reduction in hearing thresholds. ABR results were also slightly improved in the RSV 100 mg/kg group, but neither molecular-level changes nor hearing thresholds were evidently better than those in the elderly group. Previous evidence has demonstrated otoprotective effect at low to moderate doses of RSV, which was consistent with our findings (García-Alcántara et al., 2018; Su et al., 2019; Xiong et al., 2019). Additionally, Lee et al. revealed that RSV can mediate ototoxicity at high doses (Lee et al., 2020). Unsurprisingly, in our study, surface preparation data depicted elevated loss of OHC and IHC and increased disorientation in the in the RSV 300 mg/kg group. SEM data further confirmed that the damage of OHCs in the RSV 300 mg/kg group was more severe, including the loss and morphological changes of OHCs. Collectively, the above data suggested that low-dose RSV evidently reduced the ABR thresholds, hair cell loss during the progression of ARHL, and conversely increased the number of OHCs in the OC.

Necroptosis triggers inflammation and cell death. Data have confirmed that inhibition of necrotic apoptosis enhances neuroprotection, so necrotic apoptotic factors may be promising therapeutic targets (Zhang et al., 2017). The most defined molecular pathway of necroptosis is mediated through RIPK1/RIPK3/MLKL pathway. Among them, RIPK3 is a specific factor that acts as a molecular switch, and is considered essential for necroptosis, while RIPK1 is not. For MLKL, it was is a key downstream target of RIPK3 in the necrotic apoptosis pathway (Lu et al., 2018). Choi M et al. reported that RIP3-dependent necroptosis was highly expressed in cisplatin-induced ototoxicity, and the susceptible regions within the cochlea were the OCs and SGNs (Choi et al., 2019). Wang et al. showed that RIP3 was essential for mediating necroptosis in ouabain-induced SGNs damage, targeting RIP3 may prevent SGNs from death in clinical practice, and finally help the treatment of sensorineural hearing loss (Wang et al., 2021). However, few studies have investigated the role of necroptosis in the cochlea, only the study of Lyu, et al. provided the first evidence that the aging cochlea exhibits necroptosis *in vivo* (Lyu et al., 2020). Here, importantly, we further conformed that aging cochleae underwent necroptosis. Moreover, our results showed that the levels of RIPK3, RIPK1 and MLKL were promoted during progression of ARHL. Previous, Hu et al. revealed that RSV suppresses necroptosis in H9c2 cells, which may realize through the inhibition of the TNF-α/RIP1/RIP3/MLKL signaling pathway (Hu et al., 2021). Consistently, our data depicted that the protein expressions of RIPK1, RIPK3, and MLKL in cochlea tissues were sharply decreased after administration of 50 mg/kg RSV. Taken together, we proved that low-dose RSV inhibited RIPK3-mediated necroptosis in aging cochlea.

## Data Availability

The original contributions presented in the study are included in the article/supplementary materials, further inquiries can be directed to the corresponding author.
